# Remote usage of digital patient-reported outcome measures for shoulder and elbow pathologies: a systematic review

**DOI:** 10.1016/j.xrrt.2026.100795

**Published:** 2026-06-15

**Authors:** Niclas Lutz, Nils Käßer, Julius David, Felix Öttl, Rony-Orijit Dey Hazra, Asimina Lazaridou, Markus Scheibel, Claudio Rosso, David Alexander Back

**Affiliations:** a Center for Musculoskeletal Surgery, Charité – Universitätsmedizin Berlin, Corporate Member of Freie Universität Berlin, Humboldt-Universität zu Berlin, and Berlin Institute of Health, Berlin, Germany; b University of Lübeck, Lübeck, Schleswig-Holstein, Germany; c Department of Orthopedic Surgery, Balgrist University Hospital, University of Zurich, Zurich, Switzerland; d Department for Shoulder and Elbow Surgery, Schulthess Clinic, Zurich, Switzerland; e Department of Anesthesiology, Brigham & Women's Hospital and Harvard Medical School, Boston, MA, USA; f Shoulder and Elbow Unit, ARTHRO Medics AG, Basel, Basel-Stadt, Switzerland; g University of Basel, Basel, Basel-Stadt, Switzerland

**Keywords:** ePROMs, Digital-PROMs, PROMIS, CAT, EMCAT, EMA, Shoulder, Elbow

## Abstract

**Background:**

Patient-reported outcome measures (PROMs) report patient-centered outcomes. Digital PROMs enable remote monitoring of larger cohorts and support clinical decision-making without in-person visits. This review evaluates their use in shoulder and elbow pathologies, highlighting key benefits and challenges.

**Methods:**

A systematic PubMed Medical Subject Headings search (through March 2025) identified studies evaluating digital PROMs applicable outside clinical settings. Studies were screened and analyzed according to Preferred Reporting Items for Systematic Reviews and Meta-Analyses (PRISMA) guidelines.

**Results:**

The search yielded 2,331 records; 474 remained after filtering. After screening, 62 full texts were reviewed, and 11 met all inclusion criteria. Six studies focused on shoulder conditions, two on elbow pathology, and three on broader upper-extremity disorders. Study designs included six prospective cohorts, two randomized trials, two retrospective analyses, and 1 implementation study. Digital PROM tools fell into five categories and PROMs into five outcome domains. Response rates were reported in only four studies and varied substantially from 43% to 91.6%, limiting the consistency and interpretability of adherence-related findings. Baseline PROMs were collected in 8 of 11 studies. Digital tools demonstrated feasibility and patient acceptance, with comparable performance to paper-based or conventional methods only when directly assessed in individual studies.

**Conclusion:**

Electronic PROMs appear to be a feasible and acceptable option for remote follow-up in shoulder and elbow pathologies. In studies with direct comparison, their performance was generally comparable to paper-based or conventional collection methods. However, the current evidence is heterogeneous, response rates were inconsistently reported, and important barriers remain for older adults and patients with limited digital access or literacy.

In recent years, the use of patient-reported outcome measures (PROMs) has gained increasing interest in the context of postoperative quality assurance. Integrating patients' subjective perspectives following surgical interventions, and subsequently adapting treatment strategies, may enhance patient satisfaction and lead to improved clinical outcomes.^2^^,^^40^

Acute and chronic pathologies of the shoulder and elbow have a negative impact on patients' quality of life and are frequently associated with substantial limitations in performing activities of daily living. Moreover, pathologies requiring surgical intervention are frequently associated with prolonged postoperative rehabilitation.^4^^,^^34^ Given the prolonged rehabilitation periods required to regain full functional capacity after shoulder and elbow surgery, the standardized application of PROMs may provide valuable support in guiding therapy and simplifying clinical decision-making for both surgeons and patients.^10^^,^^24^ Especially in rural settings, where access to medical care often requires considerable travel distances, the use of PROMs—particularly through digital data capture in the home environment constitutes a promising alternative to conventional in-person follow-up consultations.^13^^,^^33^^,^^38^

The implementation of PROMs continues to present organizational challenges for hospitals and outpatient practices. In particular, paper-based data collection imposes additional workload within already strained clinical workflows, which may result in resistance among health-care providers and, ultimately, suboptimal or incomplete data acquisition.^25^^,^^29^ A substantial simplification of PROM collection, and thereby a broader and more comprehensive implementation, could be achieved through the advancement and widespread adoption of digital tools.^18^^,^^37^

The objective of this systematic review is to identify patient-reported outcome measures (PROMs) that are currently available in digital formats for use in shoulder and elbow surgery, to describe their modes of administration (eg, dedicated tools, web-based applications, or integration into hospital/patient information systems), to examine potential barriers to their implementation, and to discuss perspectives for further optimization. A particular focus will be placed on digital assessment methods that can be utilized by patients in their home environment and without the support of health-care professionals.

## Materials and methods

This study was designed as a systematic review (Level I evidence according to the Oxford Center for Evidence-Based Medicine classification).

### Search strategy

A systematic literature search was conducted on PubMed (up to March 2025) to identify studies reporting on digital patient-reported outcome measures (PROMs) in shoulder and elbow surgery. The search strategy combined Medical Subject Headings (MeSH) terms and free-text terms related to digital PROMs (eg, *ePROMs, electronic PROMs, digital health questionnaires, Patient-Reported Outcomes Measurement Information System (PROMIS), Computerized Adaptive Testing (CAT), Ecological Momentary Assessment (EMA)*) and shoulder or elbow conditions (eg, *shoulder surgery, shoulder joint, elbow surgery, elbow joint, upper extremity*). Terms related to the hand, wrist, or nondigital PROMs were excluded. The complete search strategy is provided in appendix. Final search criteria were as follows: ((Digital Patient Reported Outcome Measures OR ePROMs OR Online Patient Reported Outcomes OR Digital health questionnaires OR Electronic Patient Reported Outcome Measurement OR mHealth PROMs OR PROMs mobile application OR PROMs digital platform OR EMA OR CAT OR EMCAT) AND (shoulder surgery OR shoulder joint[MeSH] OR shoulder injury OR elbow surgery OR elbow joint[MeSH] OR elbow injury OR upper extremity[MeSH])) NOT (wrist[MeSH] OR hand[MeSH] OR analog PROMs OR PROMs without relevance to upper extremity).

### Eligibility criteria

Inclusion criteria included the following: (1) studies investigating the use, implementation, validation, or comparison of electronic patient-reported outcome measures (ePROMs), (2) adult patients with musculoskeletal conditions (shoulder and elbow pathologies), (3) digital data collection: digital or remote methods for PROM collection (e.g.online platforms, mobile applications, telemedicine systems), (4) comparative component (not mandatory): studies comparing ePROMs with traditional or paper-based PROMs, (5) quantitative clinical study designs (prospective cohort studies, randomized controlled trials, retrospective analyses, before-after studies), (6) studies published in English, and (7) full-text articles in peer-reviewed journals.

Exclusion criteria were as follows: (1) studies that did not evaluate, implement or use ePROMs; (2) nondigital data collection only: studies that focused on traditional or paper-based PROM-collection; (3) nonclinical populations: studies conducted in healthy volunteers (students, clinicians, technical users) without involvement of actual patients; (4) nonmusculoskeletal populations: more precisely patients without shoulder or elbow pathologies; (5) lack of validated outcome instruments: studies using nonvalidated, self-developed questionnaires only; (6) no original research studies including reviews, systematic reviews, meta-analyses, editorials, commentaries, protocols, and letters to the editor were excluded; (7) non–peer-reviews publications including conference proceedings, theses, dissertations, preprints, or reports without peer review; and (8) studies not published in English.

### Quality assessment of included studies using the Newcastle–Ottawa Scale

Of the included studies, only 1 scored 5 stars, the quality of the other ten studies ranged from good to very good (6-8 stars). The Comparability domain displayed the greatest variation (0-2/2), reflecting variability in how well studies controlled for confounding factors. Approximately half of the studies achieved partial comparability (1/2), while a smaller subset reached full comparability (2/2). Studies achieving the highest total NOS scores (8/9) primarily focused on PROMIS-based assessments, digital musculoskeletal assessment tools, or postoperative functional outcomes. Lower-scoring studies (5-6 total points) tended to involve pilot or early cohort investigations with limited participant matching or confounder control. For the detailed results see Table I.

**Table I:** Detailed results of the Newcastle -Ottawa Scale for each study.

Authors/year	Study	Selection	Comparability	Outcome	Total NOS
Viven J, Prkač T, Bjørnstad KLM, Engstrand C, 2016^41^	Effect of introducing an online system on the follow-up of elbow arthroplasty	2/4	1/2	3/3	6
Pines Y, Gordon D, Allen M, Kwon W, Zuckerman J, Virk M, 2022^32^	Performance and responsiveness to change of PROMIS UE in patients undergoing total shoulder arthroplasty	4/4	1/2	3/3	8
Kaat AJ, Li S, Berndt S, et al, 2017^22^	Longitudinal validation of the PROMIS physical function item bank in upper extremity trauma	4/4	1/2	3/3	8
Hong JH, Kim MK, Park JS, Bae DS, 2021^17^	The reliability of remote patient-reported outcome measures via mobile apps after rotator cuff repair surgery	4/4	1/2	3/3	8
Janela D, Costa F, Molinos M, et al, 2023^21^	Fear avoidance beliefs in upper-extremity musculoskeletal pain conditions: secondary analysis of a prospective clinical study on digital care programs	4/4	1/2	3/3	8
Janela D, Costa F, Molinos M, et al, 2022^19^	Digital rehabilitation for elbow pain musculoskeletal conditions: a prospective longitudinal cohort study	3/4	0/2	3/3	6
Janela D, Costa F, Molinos M, et al, 2022^20^	Asynchronous and tailored digital rehabilitation of chronic shoulder pain: a prospective longitudinal cohort study	3/4	0/2	3/3	6
Havermans RM, Lansink KW, Gosens T, van Riet RP, 2023^14^	Comparing PROMIS CAT with existing measures after operative interventions for extremity fractures	4/4	1/2	3/3	8
Fabres Martín J, Ventura Palacios C, Sanz Ruiz P, et al, 2023^6^	Telemedicine approach for patient follow-up after total knee and reverse total shoulder arthroplasty: a pilot study	2/4	2/2	3/3	7
Pak S, Janela D, Freitas A, et al, 2023^31^	Comparing digital to conventional physical therapy for chronic shoulder pain: randomized controlled trial	2/4	2/2	3/3	7
Hessburg L, Zbeda L, Arciero R, et al, 2021^15^	Patients with preoperative clinical depression symptomology experience significant improvements in postoperative PROMIS outcomes following rotator cuff repair	1/4	1/2	3/3	5

*PROMIS*, Patient-Reported Outcomes Measurement Information System; *PROMIS UE CAT*, PROMIS upper extremity computerized adaptive testing.

### Data extraction

The reviewers used a pilot-tested template and extracted data from the included studies using a standardized Microsoft Excel sheet (version 2,505; Microsoft Corp., Redmond, WA, USA). In addition, standardized prompts were developed for GPT-5 (OpenAI, San Francisco, CA, USA) to assist in title and abstract screening, full-text review, and data extraction (see Supplement 1 for Structured Data Extraction Template). GPT-5 was employed exclusively as a supportive tool to structure and summarize information; all extracted content was subsequently reviewed, and verified, by the reviewers. The extracted data items included key study characteristics (see Table II). The methodological quality and risk of bias of the included studies were assessed using the Newcastle–Ottawa Scale (NOS), which evaluates study quality in terms of selection, comparability, and outcome. As the NOS assigns a maximum of nine stars and does not specify official cutoff values, studies are commonly classified as poor quality (0-3 stars), fair quality (4-5 stars), good quality (6-7 stars), and very good quality (8-9 stars).

**Table II:** Data extraction from included studies via GPT-5 (OpenAI, San Francisco, CA, USA).

Subcategory	Focus/description	Corresponding header(s)
Bibliographic information	Identifying information about study source	Title; authors and yr; link to PDF/DOI
Clinical context	Anatomical or patient population characteristics	Body region; clinical setting & country – target patient group
PROMs (digital)	Characteristics of digitally collected PROMs	PROMs (digital acquisition); digital tool/platform
PROMs (traditional controls)	Comparator or baseline nondigital PROMs	Control-PROMs (traditional, nondigital)
Study methodology	How the research was structured	Study design; methods (digital-PROMs relevant)
Timing & follow-up	Temporal structure of data collection	Follow-up time points for PROM collection
Administration	How PROMs were delivered to participants	Administration – how PROMs were administered
Response metrics	Completion rates and burden	Response rate (digital); response burden (PROMIS)
Measurement quality	Indicates sensitivity & measurement load	Floor/ceiling effects reported?
System integration	Degree of use in clinical workflows	Integration in a clinical system?
Study aim	Context and intention	Introduction (aim/context)
Results	Key findings related to digital PROMs	Results (digital-PROMs relevant)
Interpretation	Strengths, weaknesses, feasibility	Discussion (feasibility/advantages/barriers)
Overall implications	Main takeaways	Conclusion (key takeaways)
Eligibility	Final decision for study inclusion	Inclusion (meets criteria?)

*PROMs*, patient-reported outcome measures.

### Data synthesis

Due to substantial heterogeneity in study design, patient populations, PROM instruments, digital platforms, availability of baseline data, follow-up schedules, and response-rate reporting, a formal meta-analysis was not considered appropriate. Findings were therefore synthesized narratively.

### Registration

This systematic review was prospectively registered in the PROSPERO database (ID number: 1287713).

## Results

### Study selection and inclusion

The initial PubMed search yielded 2,331 records. After refinement and filtering, 474 studies were identified. Title and abstract screening were performed independently by two reviewers, resulting in 62 articles selected for full-text review. To facilitate a more detailed analysis of the 62 selected studies, custom prompts were developed for use with GPT-5 to assist in extracting relevant content (Supplement 1).

**Figure 1:**
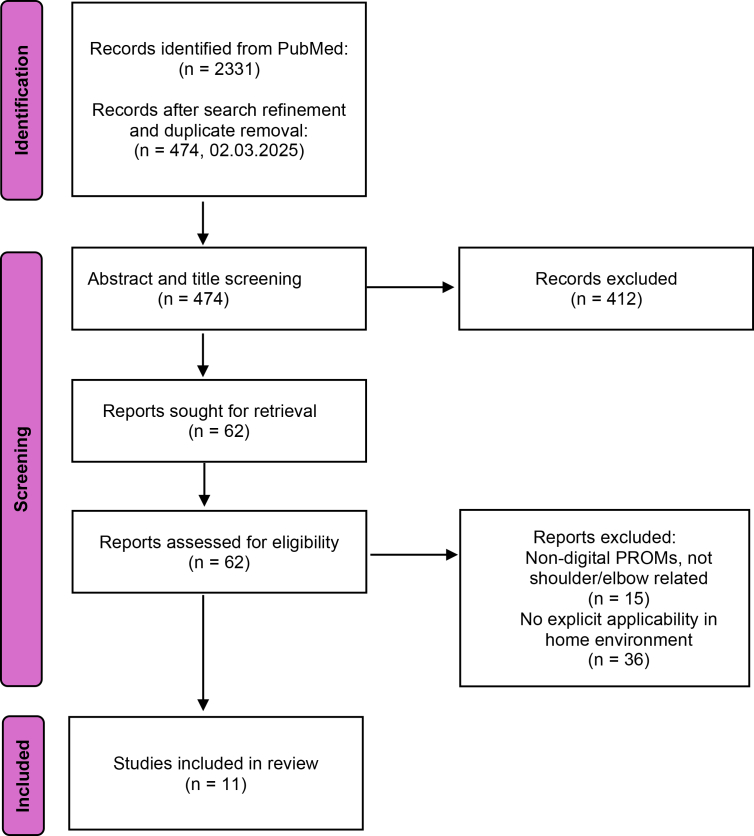
PRISMA-diagram, study selection after PubMed search. *PRISMA*, Preferred Reporting Items for Systematic Reviews and Meta-Analyses.

The results and information extracted using GPT-5 were individually verified for accuracy by the reviewers. GPT-5 was used solely for information summarization. During full-text screening, 15 studies were excluded due to nondigital PROM collection or nonsolely shoulder/elbow patient populations. An additional 36 studies were excluded because they did not explicitly report PROM administration applicable in a remote (nonclinical) environment. Finally, 11 studies met all inclusion criteria and were included in the final analysis. The study selection process is summarized in the Preferred Reporting Items for Systematic Reviews and Meta-Analyses (PRISMA) flow diagram (Fig. 1).

### Characteristics of included studies

Of the included studies, six focused exclusively on shoulder pathologies, two on elbow pathologies, two on upper extremity (shoulder, elbow, hand/wrist) and 1 on shoulder/elbow as well as lower extremity (total knee arthroplasty), which was not taken into consideration for this study.

The study designs included six prospective cohort studies (single-arm, longitudinal, multicenter, and test-retest reliability), two randomized controlled trials, two retrospective/secondary analyses (retrospective comparative study and secondary analysis of a prospective trial), and 1 before-after (implementation) study.

### Digital tools and administration

The digital tools and their modes of administration could be clustered into five categories. Web/E-mail survey platforms (remote, link-accessed), Tablet in clinic, with home access via Web portal or E-Mail, Standalone mobile app, Digital rehabilitation platforms with sensors and remote monitoring (SWORD Health, Firma, Ort, Land) and asynchronous telemedicine (store-and-forward) platforms. Table III contains detailed informations about the used digital tools.

**Table III:** Digital tools for data collection.

	Digital rehabilitation platforms + remote monitoring	Web/E-mail survey platforms	Tablet in clinic with home web/E-mail access	Standalone mobile app	Asynchronous telemedicine (store-and-forward) platforms
Category	Digital rehabilitation platforms + remote monitoring	Web/E-mail survey platforms (remote link-accessed)	Tablet in clinic with home access via web portal or E-mail	Standalone mobile app	Asynchronous telemedicine (store-and-forward) platforms
Administration & setting	Fully digital rehab with monitoring and clinician dashboard	Remote via Web links or E-mail on computer/smartphone	In-clinic tablet + secure home completion	In clinic and at home via smartphone app	Remote review of uploaded data/models
Studies (n)	4	3	2	1	1
Platforms/tools mentioned	SWORD Health	REDCap, OnlinePROMS, Qualtrics BV, E-mail-based surveys	Assessment CenterSM (HealthMeasures), REDCap	Proscore (Incheon, South Korea)	AlmaHealthPlatform with patient-specific model (PSM)
Workflow summary	Postoperative rehab surveillance with patient-guided exercises, therapist dashboard, embedded PROMs	Patients receive a link by E-mail; complete PROMs remotely	In clinic: tablet capture; if incomplete, customized URL/E-mail enables secure home completion; research assistant follows up for missing forms	App triggers notifications; home entries via mobile messages; all PROMs completed within the app	PSM includes ROM, functional scores, radiographs for asynchronous assessment
Data handling/integration	Platform-native capture; centralized monitoring and logging	Direct entry to REDCap or OnlinePROMS; data stored centrally	Data stored in REDCap/platform; secure participant accounts/links	Electronic PROM system within Proscore; centralized storage	Data packaged into PSM; store-and-forward within platform

*PROMs*, patient-reported outcome measures; *ROM*, range of motion.

### Electronic patient-reported outcome measures

The digitally acquired PROMs in the included studies were also grouped into the following five subcategories: psychological/biopsychosocial and work-related PROMs, upper-extremity/region-specific functional measures, PROMIS domain CATs (upper extremity, function, pain, and mood), pain intensity measures, and generic health–related quality of life PROMs. Table IV contains the used ePROMs as well as more detailed information.

**Table IV:** ePROMs used in the included studies.

Subcategory	Focus/Construct	Instruments (with counts)	Notes
Psychological/biopsychosocial & work-related	Anxiety, depression, fear-avoidance, work considerations	GAD-7 (4), PHQ-9 (4), FABQ-PA (3), WAI (2)	Captures barriers to adherence beyond region-specific disability
Upper-extremity/region-specific functional measures	Shoulder/elbow/upper-limb function and symptoms	QuickDASH (4), ASES (2), DASH (1), OSS (1), SST (1)	Core region-specific scores for upper extremity; useful across mixed settings
PROMIS domain CATs (upper extremity, function, pain, mood)	Computer-adaptive assessment of function, pain interference, mood	PROMIS UE CAT (3), PROMIS Physical Function CAT (2), PROMIS Pain Interference (2), PROMIS Self-Rated CAT (1), PROMIS Depression CAT (1)	Adaptive measurement reduces burden while maintaining precision
Pain intensity measures	Global pain intensity over time	NRS/NPRS (4), VAS pain (2)	High-feasibility scales; often combined with other PROMs
Generic health-related quality of life	Overall health status/utility	EQ-5D (1)	Not region-specific; useful for cost-utility and cross-condition comparisons

*ePROMs*, electronic patient-reported outcome measures; *GAD-7*, Generalized Anxiety Disorder-7; *PHQ-9*, Patient Health Questionnaire-9; *FABQ-PA*, Fear-Avoidance Beliefs Questionnaire–Physical Activity; *WAI*, Work Ability Index; *ASES*, American Shoulder and Elbow Surgeons; *DASH*, Disabilities of the Arm, Shoulder and Hand; *OSS*, Oxford Shoulder Score; *SST*, Simple Shoulder Test; *PROMIS*, Patient-Reported Outcomes Measurement Information System; *PROMIS UE CAT*, PROMIS upper extremity computerized adaptive testing; *NRS*, Numeric Rating Scale; *NPRS*, Numeric Pain Rating Scale; *VAS*, visual analog scale; *EQ-5D*, EuroQol-5 Dimensions.

### Follow-up time points

Most studies established a baseline before the intervention or surgery (8/11). About half of the studies (5/11) then captured an early change within 2-12 weeks, often including a 3-month/12-week visit. The same number (5/11) reported a 6-month outcome, which was the most consistent midterm time point. Long-term outcomes at 12 months or later were also reported in 5/11 studies, although only 1 study continued structured follow-up beyond 1 year (up to 10 years). Three studies deviated from calendar-based schedules and used program-bound follow-up at 8 weeks. Because follow-up intervals ranged from early postoperative assessments to long-term follow-up, the outcome data were not considered directly comparable across studies.

Detailed follow-up time points of the included studies can be found in Table V.

**Table V:** Overview of included Studies including follow-up time points and NOS.

Authors	Year	Journal	Title	ePROMs	Traditional PROMs for comparison	Follow-up time points	Total NOS
Viven J, Prkač T, Bjørnstad KLM, Engstrand C^41^	2016	World Journal of Orthopedics	Effect of introducing an online system on the follow-up of elbow arthroplasty	Oxford Elbow Score (OES), EQ-5D, VAS pain	Paper-based OES/EQ-5D/VAS	Preoperative, 1, 3, 5, 7, 10 yr postoperative	6
Pines J, Gordon D, Allen M, Kwon W, Zuckerman J, Virk M^32^	2022	Journal of Orthopedic Research	Performance and responsiveness to change of PROMIS UE in patients undergoing total shoulder arthroplasty	PROMIS UE CAT v2.0	ASES, Oxford Shoulder Score (OSS), Simple Shoulder Test (SST)	Pre-op, 6 weeks, 12 weeks, 6-12 mo postop	8
Kaat AJ, Li S, Berndt S, et al^22^	2017	Journal of Orthopedic Trauma	Longitudinal validation of the PROMIS physical function item bank in upper extremity trauma	PROMIS PF CAT v1.2	None	Baseline (≤12 mo post-treatment) and 6 mo later	8
Hong JH, Kim MK, Park JS, Bae DS^17^	2021	Journal of Medical Internet Research	The reliability of remote patient-reported outcome measures via mobile apps after rotator cuff repair surgery	ASES scale (electronic), DASH	Paper-based equivalents (validated subscale)	1, 2, 3, 6, 12 mo postoperative	8
Janela D, Costa F, Molinos M, et al^21^	2023	Pain Medicine	Fear avoidance beliefs in upper-extremity musculoskeletal pain conditions: secondary analysis of a prospective clinical study on digital care programs	QuickDASH (digital), FABQ-PA, NRS pain, GAD-7, PHQ-9	None	Baseline, end of program (∼8 weeks)	8
Janela D, Costa F, Molinos M, et al^19^	2022	International Journal of Environmental Research and Public Health	Digital rehabilitation for elbow pain musculoskeletal conditions: a prospective longitudinal cohort study	QuickDASH (digital), NRS pain, PHQ-9	None	Baseline, end of 8-week intervention	6
Janela D, Costa F, Molinos M, et al^20^	2022	Journal of Pain Research	Asynchronous and tailored digital rehabilitation of chronic shoulder pain: a prospective longitudinal cohort study	QuickDASH (digital), NPRS pain, GAD-7, PHQ-9, FABQ-PA, WAI	None	Baseline, 12 weeks	6
Havermans RM, Lansink KW, Gosens T, van Riet RP^14^	2023	Value in Health	Comparing PROMIS CAT with existing measures after operative interventions for extremity fractures	PROMIS PF CAT, PROMIS PI CAT, PROMIS Depression CAT	QuickDASH, LEFS	Week 2, Week 6, mo 3, mo 6 postop	8
Fabres Martín J, Ventura Palacios C, Sanz Ruiz P, et al^6^	2023	International Journal of Computer Assisted Radiology and Surgery	Telemedicine approach for patient follow-up after total knee and reverse total shoulder arthroplasty: a pilot study	ASES, SST (digitally collected via telemedicine software)	Face-to-face completion of same PROMs	Single follow-up (post-operative, 2018–2019 surgeries)	7
Pak S, Janela D, Freitas A, et al^31^	2023	Journal of Medical Internet Research	Comparing digital to conventional physical therapy for chronic shoulder pain: randomized controlled trial	QuickDASH (short-form, digital), PHQ-9, GAD-7, NPRS	Conventional in-person PT using same PROMs	Baseline, Week 4, Week 8	7
Hessburg L, Zbeda L, Arciero R, et al^15^	2021	Arthroscopy: The Journal of Arthroscopic & Related Surgery	Patients with preoperative clinical depression symptomology experience significant improvements in postoperative PROMIS outcomes following rotator cuff repair	PROMIS UE CAT, PROMIS PI CAT, PROMIS Depression CAT	None	Baseline, 6 mo, ≥1 yr post-operative	5

*PROMIS*, Patient-Reported Outcomes Measurement Information System; *PROMIS UE CAT*, PROMIS upper extremity computerized adaptive testing; *PROMIS PF CAT*, PROMIS physical function computerized adaptive test; *PROMIS PI CAT*, PROMIS pain interference computerized adaptive test; *NOS*, Newcastle-Ottawa Score; *ASES*, American Shoulder and Elbow Surgeons; *ePROMs*, electronic patient-reported outcome measures; *PROMs*, patient-reported outcome measures; *EQ-5D*, EuroQol-5 Dimensions; *VAS*, visual analog scale; *DASH*, Disabilities of the Arm, Shoulder and Hand; *NRS*, Numeric Rating Scale–Pain; *GAD-7*, Generalized Anxiety Disorder-7; *PHQ-9*, Patient Health Questionnaire-9; *FABQ-PA*, Fear-Avoidance Beliefs Questionnaire–Physical Activity; *WAI*, Work Ability Index; *LEFS*, Lower Extremity Functional Scale.

### Response rates and burden

Only four of 11 studies reported response rates. One implementation study of an online PROM system achieved a response rate of 91.6% after sending 143 questionnaires to patients. A fully digital, home-based rehabilitation program reported a completion rate of 84.4% (112/132). A similar home-based physical therapy study reported a response rate of 79.1% with 296 patients enrolled (234/296). In a longitudinal upper-extremity cohort with a 6-month follow-up, the response rate was 43%: 424 patients completed baseline forms during in-person visits, 307 consented to be recontacted, and 132/307 completed PROMs at 6 months.

The remaining 7 studies reported sample sizes without percentage response rates. A prospective shoulder-surgery study recorded 96 completed PROMs. Another study enrolled 174 patients, of whom 59 provided additional PROM data during a remote 12-month follow-up. A larger study evaluating a digital rehabilitation program via PROM collection included 520 participants. In an email-based follow-up after upper-extremity fractures, 259 patients completed online surveys. A telemedicine feasibility study of postoperative follow-up after total knee and reverse shoulder arthroplasty included 12 shoulder patients. A randomized trial comparing conventional vs. digital physiotherapy analyzed 82 patients. The final study assessed PROMIS outcomes after rotator cuff repair in 340 patients, including 65 with preoperative depression.

Across the included studies, respondent burden was rarely quantified. Use of PROMIS CAT or app-based questionnaires was generally described as feasible and easy to complete. An automated, E-Mail-based online PROM system was also reported to be completed consistently. None of the 11 studies provided a standardized quantitative measure of respondent burden (eg, time to completion, number of items, or perceived effort).

### Key findings relevant to digital patient-reported outcome measures

Viveen et al^41^ reported that implementing a structured online follow-up system may increase response rates and completeness; however, early results may be biased by intensified collection efforts, and longer follow-up is needed. Pines et al^32^ found that PROMIS upper extremity computerized adaptive testing (PROMIS UE CAT) produced results comparable to legacy PROMs and demonstrated meaningful responsiveness as early as 6 weeks after shoulder arthroplasty. Kaat et al^22^ described PROMIS CAT as reliable, responsive, and valid, with the added benefit of reducing patient completion time. Hong et al observed that mobile app–based remote PROM collection yields results comparable to in-clinic collection from 6 months onward; earlier time points showed significant differences. They also noted potential barriers for older adults and recommended alternatives (eg, paper-based PROMs).^17^ Janela et al^21^ reported very high adherence, completion rates, and patient satisfaction after implementing a home-based digital care program, including among patients with high fear-avoidance beliefs, underscoring the value of multimodal, digitally delivered care. Similar findings from two additional studies by Janela et al^19^^,^^20^ showed high engagement, completion, and satisfaction with digital care programs for elbow and shoulder pain. Havermans et al^14^ evaluated PROMIS CAT after operative treatment of upper-extremity trauma and found it efficient with results comparable to legacy PROMs; they emphasized the need for integration into clinic software to avoid excessive collection burden. Fabrés Martín et al^6^ assessed an asynchronous telemedicine tool to obtain remote range of motion measurements after reverse shoulder arthroplasty; the tool received high user ratings, reduced follow-up time, and showed good concordance with in-clinic measurements. Pak et al reported that digital physical therapy programs achieved high acceptance and satisfaction, with clinical outcomes comparable to conventional physiotherapy in that individual randomized trial. Because this was the only included study with this specific comparison, its findings were not suitable for subgroup analysis and were considered descriptively within the narrative synthesis.^31^ Hessburg et al^15^ demonstrated significant postoperative improvements in PROMIS CAT scores after rotator cuff repair even among patients with preoperative depression; however, exclusion of non–English-speaking patients and those unable to operate a tablet highlighted access barriers to digital PROM collection.

## Discussion

The most important finding of this systematic review was that ePROMs appear feasible and acceptable for remote follow-up in shoulder and elbow pathologies. However, only four included studies reported response rates, and these ranged from 43% to 91.6%. In addition, baseline data, follow-up schedules, and reporting standards varied substantially across studies. Accordingly, the present review supports feasibility and context-specific comparability rather than a general claim of superiority of electronic over conventional PROM collection.

The published literature across different medical fields showed similar results in terms of the utilization of ePROMs. A study involving oncology patients demonstrated that the introduction of ePROMs improved the detection of previously under-recognized physical and psychological symptoms and was associated with high patient acceptance.^42^ Malhotra et al^26^ evaluated fully ePROM collection in an orthopedic outpatient clinic and reported a participation rate of approximately 86%, concluding that electronic collection is both feasible and well accepted in the ambulatory setting. De Faoite et al^5^ analyzed the use of ePROMs after total knee arthroplasty and found similar results, including an efficient workflow, high data completeness, and the possibility of remote monitoring. Gani et al^7^ reported that the use of ePROMs during radiotherapy for oncology patients positively influenced patients' perception of clinician empathy, even though consultation time and eye contact were reduced compared with traditional PROM collection. Trojan et al^39^ observed lower hospitalization rates and fewer unplanned visits among oncology patients receiving systemic therapy when monitored digitally, compared with a retrospective control cohort, underscoring the clinical value of digital monitoring. Willinge et al^43^ described web-based and mobile-supported ePROMs as feasible, associated with high patient satisfaction, and capable of conserving clinical resources; however, effective integration into hospital information systems and structured staff training are essential for successful implementation. Hamilton et al^12^ highlighted that implementing ePROMs and enabling home-based data collection may improve shared decision-making, management of patient expectations, and overall follow-up care. Comparing traditional PROMs and nondigital collection methods, the literature shows similar results. Gwaltney et al,^11^ in a meta-analysis of 46 studies, reported strong agreement between electronic and paper-based PROMs, with equivalent outcomes. Muehlhausen et al^28^ demonstrated in a systematic review that no clinically relevant differences exist between paper and electronic formats, provided that ePROMs are properly administered and validated. Griffiths-Jones et al^9^ explicitly compared web-based remote PROM collection with paper-based methods after hip surgery and also concluded that the results were similar. Comparable findings have been reported in studies in palliative care,^1^ internal medicine chronic obstructive pulmonary disease,^30^ and hematologic oncology.^8^ In the shoulder and elbow studies included in the present review, however, direct comparisons between digital and conventional PROM collection were available only in selected study contexts. Therefore, these findings support context-specific comparability rather than universal equivalence or superiority of ePROMs.

At the same time, several reviews have also identified disadvantages and barriers associated with ePROM collection. Meirte et al noted disadvantages for older adults and for patients without access to computers or the internet, as well as the potential under-representation of certain demographic groups (lower income, lower educational level). They also highlighted technical problems, the additional workload required to maintain IT infrastructure, and the need to comply with strict data protection regulations.^27^ In addition to the dependence on technology and increased workload for staff—particularly when PROM collection is not well integrated into electronic health record systems—Salmani et al^36^ emphasized the risk of higher dropout rates if patients feel overwhelmed by digital interfaces. Consolo et al^3^ further mentioned that interacting with a screen instead of a clinician may compromise the doctor–patient relationship. In summary, the most frequently reported challenges of digital PROM collection include the specific barriers faced by certain patient groups (older adults, socially disadvantaged individuals, those with cognitive impairment, limited digital literacy, or severe illness), technical issues (IT infrastructure limitations, system failures, connectivity problems), increased workload due to poor system integration, and the potential compromise of the personal doctor–patient interaction.^16^^,^^23^^,^^29^^,^^35^ Nevertheless, further investigations and comparative studies are required, and improved integration into various practice and hospital information systems is needed to minimize additional workload associated with data collection. Moreover, the success of digital data collection depends on the patients' computer literacy and may lead to data-entry difficulties, particularly in older patients.

This study had several limitations. The search was limited to PubMed; studies not published in peer-reviewed journals or indexed in other databases might have been overlooked. Only studies reporting digital PROM administration in the treatment of shoulder or elbow pathologies were included, which restricts generalizability and may underestimate the broader applicability of ePROMs. Due to substantial heterogeneity of the included studies in terms of study design, digital tools used, PROM types, follow-up schedules, and reporting standards, the direct comparability was limited. Furthermore, the response rates and patient burden were not consistently reported, making a robust assessment of feasibility across studies difficult. Some included studies only provided sample sizes without denominator data, limiting interpretation of adherence. Another limitation is the rapidly evolving landscape of digital health technologies; newer platforms may not yet be represented in the current literature. Given the substantial heterogeneity in study design, patient populations, timing of outcome assessment, and reporting practices, a formal quantitative synthesis was not considered appropriate.

Finally, the use of GPT-5 (Open AI, CA, USA) to assist with data extraction introduces a potential source of information extraction bias, although all extracted content was manually checked for accuracy and systematically validated. These limitations should be considered when interpreting the findings of this study.

Future work should focus on the standardization and optimization of digital PROM implementation in orthopedics and on its transferability to other medical fields. In particular, future studies should examine which engagement strategies best support sustained patient participation and long-term response completeness. To ensure equitable use of ePROMs across diverse patient populations, strategies to support older adults, socioeconomically disadvantaged groups, and individuals with limited digital literacy are essential. Simplified user interfaces, multilingual options, hybrid digital-paper workflows, and better integration into existing clinical software systems will be critical for reducing administrative burden and improving implementation.

## Conclusion

ePROMs appear to be a feasible and acceptable option for remote follow-up in shoulder and elbow pathologies. In studies with direct comparison, their performance was generally comparable to paper-based or conventional collection methods. However, the available evidence is heterogeneous, response rates were inconsistently reported, and relevant barriers remain for older adults and patients with limited digital access or literacy. Therefore, current evidence supports selective implementation rather than a general claim of superiority, and further standardized comparative studies are needed.

## Disclaimers:

Funding: The study was supported by the German, Austrian and Swiss Shoulder and Elbow Society (DVSE).

Conflicts of interest: The authors, their immediate families, and any research foundations with which they are affiliated have not received any financial payments or other benefits from any commercial entity related to the subject of this article.

## Declaration of Generative AI and AI-Assisted Technologies in the Writing Process

During the preparation of this work, the authors used GPT-5 (OpenAI, San Francisco, CA, USA) to assist with data extraction during the full text screening. After using these tools/services, the authors reviewed and edited the content as needed, verified all data extraction, and take full responsibility for the content of the publication.
