# Supplementary material for: Remote usage of digital patient-reported outcome measures for shoulder and elbow pathologies: a systematic review
Source: JSES Rev Rep Tech. 2026 Jun 15;6(4):100795. doi: 10.1016/j.xrrt.2026.100795 (PMC13380199; doi:10.1016/j.xrrt.2026.100795)
Supplement: Supplementary [file mmc1.docx]

**Supplement 1**

Structured Data Extraction Template – ePROMs in Shoulder & Elbow Surgery for ChatGPT-5:
You are an expert in systematic reviews focused on orthopedic surgery and digital health. Please extract and summarize the following study according to the structured format below. Focus exclusively on the digital collection of established Patient-Reported Outcome Measures (PROMs) in shoulder and/or elbow surgery. Do not include studies that involve wrist/hand surgery or analog/paper-based PROMs. Important: Focus exclusively on information related to the digital collection of established PROMs in shoulder and/or elbow surgery. Ignore general PROM information, studies involving wrist/hand surgery, or paper-based (analog) PROMs. Extract the following data from the study: Title; Authors and year; Link to PDF / DOI; Body region (Shoulder / Elbow / Both); PROMs (digital acquisition - which PROMs were collected digitally (e.g., PROMIS PF, QuickDASH) ; Digital Tool / platform (what tool or platform was used for digital collection (e.g., PROMIS CAT, email-based, app); Control-PROMs (Which traditional (non-digital) PROMs were compared (e.g., QuickDASH, LEFS); Study design (e.g., Prospective cohort, RCT); Clinical setting and country -Target patient group (e.g., Adults 18–75 post upper extremity surgery); Follow-up time points for PROM collection (e.g., Week 2, Month 3, etc.); Administration; How PROMs were administered (e.g., Email link, app, tablet at clinic); Response Rate (Reported response rates for digital PROMs); Response Burden (PROMIS) (Number of questions, burden on patient); Floor/Ceiling Effects?; Were these reported? (Yes/No/Not stated); Integration in a clinical system?; Was the PROM tool integrated into EMR/clinic software? (Yes/No/Unclear) -Introduction (Summary of aim and context); Methods (Summary of methodology relevant to digital PROMs); Results (Summary of findings relevant to digital PROM collection); Discussion (Interpretation, feasibility, barriers, advantages); Conclusion (Key takeaways for clinical digital PROM use); Inclusion - Should this study be included based on criteria? (Yes/No).
